# Single-Cell Analysis of Brain Arteriovenous Malformations Reveals Pro-Angiogenic Myeloid Programs

**DOI:** 10.3390/brainsci16080811

**Published:** 2026-07-30

**Authors:** Benjamin Beyersdorf, Stefanos Voglis, Zsolt Kulcsar, Luca Regli, Menno R. Germans

**Affiliations:** 1 Department of Neurosurgery, Clinical Neuroscience Centre, University Hospital Zurich, University of Zurich, 8091 Zurich, Switzerlandmenno.germans@usz.ch (M.R.G.); 2 Department of Neurosurgery, University Hospital Frankfurt, 60590 Frankfurt am Main, Germany; 3 Department of Neuroradiology, Clinical Neuroscience Centre, University Hospital Zurich, University of Zurich, 8091 Zurich, Switzerland

**Keywords:** single-cell RNA sequencing, immune microenvironment, macrophages, angiogenesis, extracellular matrix remodeling, myeloid cells

## Abstract

**Highlights:**

**What are the main findings?**

- The immune landscape of brain arteriovenous malformations shows a trend towards higher proportions of M1-like macrophages, dendritic cells, monocytes, CD8 T cells and activated T cells compared with control brain tissue.
- Transcriptional programs related to angiogenesis are enriched in myeloid cell populations including M1-like macrophages, monocytes, and dendritic cells, implicating these immune populations as important contributors to vascular remodeling in brain arteriovenous malformations.

**What are the implications of the main findings?**

- The immune microenvironment, and particularly myeloid cell populations, may be associated with pathological vascular remodeling in brain arteriovenous malformations.
- Targeting immune-mediated angiogenic pathways could represent a potential therapeutic strategy to modulate lesion progression and instability.

**Abstract:**

**Background/Objectives**: Brain arteriovenous malformations (bAVMs) are complex cerebrovascular lesions that carry a substantial risk of intracranial hemorrhage, yet the biological mechanisms underlying vascular remodeling remain incompletely understood. As increasing evidence suggests that inflammatory processes contribute to vascular remodeling and bAVM progression, this study aimed to characterize the cellular composition of the immune microenvironment in human bAVMs at single-cell resolution and to identify immune cell populations associated with transcriptional programs related to angiogenesis and extracellular matrix remodeling. **Methods**: Publicly accessible single-cell RNA sequencing data from five human bAVM samples and five control brain specimens were analyzed. After quality control and data integration, immune cell types were annotated based on canonical marker gene expression. To assess biological processes relevant to vascular remodeling, the expression of predefined gene sets associated with angiogenesis and extracellular matrix remodeling, derived from the Molecular Signatures Database (MSigDB), was quantified across cell populations using rank-based gene set scoring (UCell). Differences between bAVM and control samples were evaluated at the sample level using the Wilcoxon rank-sum test. For each immune cell type, mean UCell scores were calculated per biological sample and compared between groups. To account for multiple comparisons across cell types, *p*-values were adjusted using the Benjamini–Hochberg procedure. **Results**: After quality control, 46,360 immune cells were analyzed. Compared with control tissue, bAVMs showed numerically higher proportions of lymphoid (activated T cells and CD8 T cells) and myeloid populations (M1-like macrophages, monocytes, and dendritic cells). Angiogenesis-related transcriptional activity was highest in myeloid cells and significantly increased in bAVMs, particularly in M1-like macrophages (UCell score 0.22 vs. 0.13), dendritic cells (0.16 vs. 0.10) and monocytes (0.20 vs. 0.15), whereas proliferating CD8 T cells showed a lower score (0.04 vs. 0.05; adjusted *p* = 0.040 for all four). Extracellular matrix-related programs showed a similar but weaker and non-significant pattern in myeloid populations (e.g., monocytes, dendritic cells, microglia-like cells; adjusted *p* = 0.09). **Conclusions**: Our findings identify myeloid cells, particularly M1-like macrophages, monocytes and dendritic cells, as important immune populations associated with angiogenesis in bAVMs. These findings highlight a potential role of immune-driven vascular remodeling in the pathophysiology of bAVMs.

## 1. Introduction

Although most brain arteriovenous malformations (bAVMs) are considered congenital vascular malformations and may remain clinically silent throughout life, they may undergo progressive vascular remodeling through pro-angiogenic processes and remodeling of the extracellular matrix (ECM) [1,2]. Increasing evidence suggests that pathological vascular remodeling is driven by pro-angiogenic signaling and extracellular matrix reorganization, but the biological mechanisms underlying these changes remain incompletely understood [3]. Inflammation has increasingly been recognized as an important contributor to vascular remodeling, with inflammatory infiltrates repeatedly observed in bAVM tissue [4]. These observations suggest that immune–vascular interactions may contribute to vascular remodeling, yet the specific immune cell populations driving these processes remain poorly defined.

Inflammation and immune-mediated vascular remodeling are well-established components of other cerebrovascular diseases, including intracranial aneurysms, atherosclerosis, and cavernous malformations [5,6,7]. In these conditions, immune cells can alter vessel integrity through various processes, including modulation of pro-angiogenic pathways and ECM remodeling [8,9]. In bAVMs, histopathological studies have reported perivascular inflammatory infiltrates and macrophage enrichment, and molecular analyses have identified activation of inflammatory and angiogenesis-related pathways [10,11]. However, most of this evidence is derived from bulk tissue analyses, which cannot resolve the full diversity of immune cell populations or define their transcriptional states within individual lesions. As a result, it remains unresolved which specific immune cell types are most closely associated with angiogenesis and ECM remodeling in bAVMs.

Single-cell RNA sequencing (scRNA-seq) provides a high-resolution technology to address this gap by characterizing immune heterogeneity and functional states within complex vascular lesions [12,13]. By profiling hundreds to thousands of cells per sample, scRNA-seq enables high-resolution molecular characterization of immune cells while simultaneously quantifying transcriptional programs related to angiogenesis and ECM remodeling.

In the present study, we aimed to characterize the immune microenvironment of human bAVMs at single-cell resolution, to quantify immune cell composition and to identify specific cell types associated with angiogenesis- and ECM remodeling-related programs. Given prior evidence implicating inflammation in vascular remodeling, we hypothesized that distinct immune cell subsets, particularly within the myeloid compartment, exhibit transcriptional programs associated with angiogenesis and ECM remodeling and may thereby contribute to vascular remodeling in bAVMs.

## 2. Materials and Methods

### 2.1. Study Design and Data Source

In this study, we analyzed publicly available scRNA-seq data from immune cells derived from (i) five human bAVM samples and (ii) five control samples retrieved from epilepsy patients undergoing temporal lobectomies [13]. The dataset originates from a single-cell transcriptomic atlas of the adult human cerebrovasculature comprising more than 180,000 cells. In that study, tissue was freshly dissociated into single-cell suspensions and processed using the 10x Genomics Chromium platform. Specifically, the immune-cell subset of this dataset was obtained from a publicly available resource: https://cells.ucsc.edu/?ds=adult-brain-vasc (accessed on 26 February 2026).

### 2.2. Ethics Statement

This study was conducted in accordance with the Declaration of Helsinki. Because the current study represents a secondary analysis of de-identified publicly available data, no additional ethical approval was required.

### 2.3. Data Quality Control

Cells were filtered based on the number of detected genes and the proportion of mitochondrial transcripts. Cells with fewer than 1000 detected genes were excluded because they likely represent damaged cells or empty droplets containing ambient RNA, whereas cells with more than 6000 detected genes, indicative of multiplets, were removed. In addition, cells with a mitochondrial transcript fraction exceeding 20% were excluded, as these typically reflect damaged cells. Potential doublets were identified using the scDblFinder package (version 1.24.10) in R (version 4.5.2) and removed. Doublet detection was performed separately for each biological sample to account for sample-specific cell compositions.

### 2.4. Normalization, Sample Integration and Clustering

Gene expression counts were normalized to account for differences in sequencing depth and technical variability. Data integration was performed using the Harmony R package (version 1.2.4) to correct for batch effects at the sample level. Cells were subsequently clustered using unsupervised graph-based shared nearest-neighbor clustering as implemented in the Seurat R package (version 5.4.0) to group transcriptionally similar cells. Marker genes for each cluster were identified using differential expression analysis to facilitate biological interpretation and manual cell type annotation.

### 2.5. Cell Type Annotation

First, clusters were annotated based on the expression of canonical lineage marker genes [14,15,16]. Broad immune lineages were identified, including myeloid and lymphoid compartments. A small cluster expressing neuronal markers was identified and excluded from subsequent immune-specific analyses. To increase annotation resolution, cells belonging to the myeloid and lymphoid compartments were analyzed separately by subsetting each lineage and performing independent reclustering, allowing the identification of transcriptionally distinct immune cell states within each compartment.

### 2.6. Functional Module Scoring

To investigate transcriptional programs related to vascular remodeling, gene sets associated with angiogenesis and ECM remodeling were obtained in an unbiased fashion from the Molecular Signatures Database (MSigDB) [17]. Gene sets were restricted to include only genes present in the expression matrix of the analyzed dataset of immune cells and those detectably expressed in at least 5% of cells. Genes not meeting these criteria were excluded from the analysis. The final angiogenesis module comprised APP, CCND2, FGFR1, ITGAV, JAG1, LPL, LRPAP1, NRP1, OLR1, PDGFA, PTK2, S100A4, SPP1, THBD, TIMP1, TNFRSF21, VAV2, VCAN, and VEGFA, while the ECM remodeling module included COL18A1, FURIN, MMP14, MMP2, MMP24, MMP9, TIMP1, and TIMP2. Gene set activity was quantified at the single-cell level using UCell, a rank-based scoring method that assesses the enrichment of predefined gene sets based on the relative expression ranks of genes within each cell [18]. 

### 2.7. Statistical Analysis

Two types of comparison were performed between bAVM and control samples, both at the level of biological samples. First, the relative proportion of each annotated immune cell population was compared between groups. Second, for each cell population the mean UCell score of the angiogenesis program and of the extracellular-matrix–remodeling program was computed per sample and compared between groups. All comparisons used the two-sided Wilcoxon rank-sum test with the normal approximation. For the smallest *p*-values this yields more conservative values than the exact test (0.0122 versus 0.0079). Within each of the three analyses (composition, angiogenesis, and ECM remodeling) separately, *p*-values were adjusted for multiple testing across the 13 immune cell populations using the Benjamini–Hochberg procedure, and an adjusted *p* < 0.05 was considered statistically significant.

## 3. Results

### 3.1. Single-Cell Profiling Reveals Immune Cell Composition in Human bAVMs

**Figure 1:**
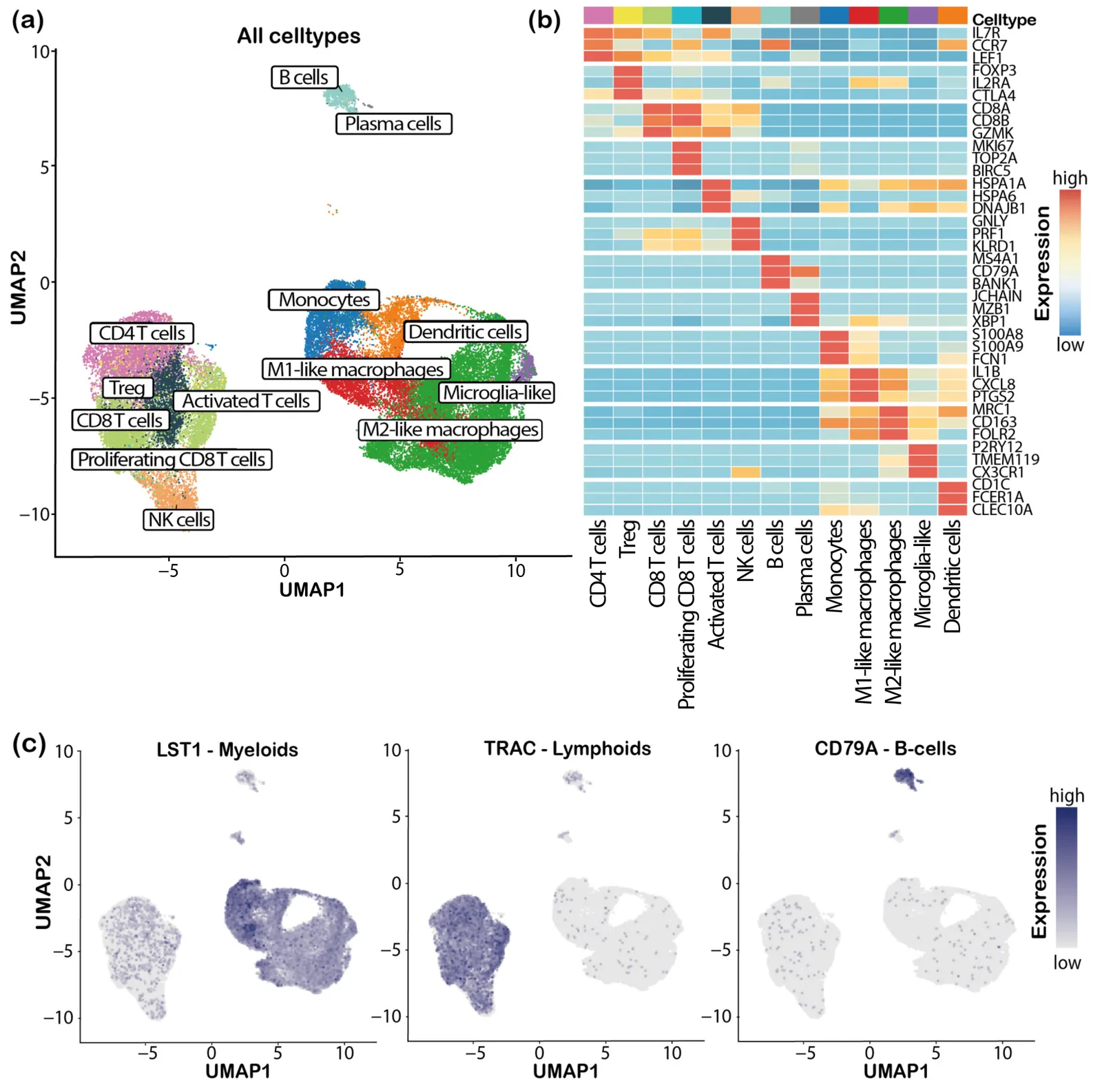
The immune microenvironment in human bAVMs. (**a**) Uniform Manifold Approximation and Projection (UMAP) of integrated immune cells from bAVM and control samples showing immune-cell annotations. (**b**) Heatmap showing average expression of selected marker genes across annotated immune-cell populations. (**c**) Feature plots showing expression of representative lineage markers, including the myeloid marker LST1, the T-cell marker TRAC, and the B-cell marker CD79A. Abbreviations: Treg, regulatory T cells.

After quality control and doublet removal, a total of 46,360 immune cells from five bAVM and five control samples were retained for downstream analysis. Unsupervised clustering and annotation of the integrated dataset identified a diverse immune landscape comprising 13 different lymphoid (e.g., T- and B-lineage cells) and myeloid (e.g., monocytes, M1-like and M2-like macrophages, dendritic cells, and microglia-like cells) cell populations. A more detailed subdivision into transcriptionally distinct immune cell populations is shown in Figure 1a. Marker-based heatmaps demonstrated clear cell type-specific transcriptional programs (Figure 1b), and representative feature plots supported lineage identity, with canonical expression of myeloid (LST1), T-cell (TRAC), and B-cell (CD79A) markers (Figure 1c). Additional analyses and marker gene visualizations supporting immune-cell annotation are provided in Supplementary Figures S1 and S2.

### 3.2. Immune Cell Composition in bAVMs and Control Brain Tissue

**Figure 2:**
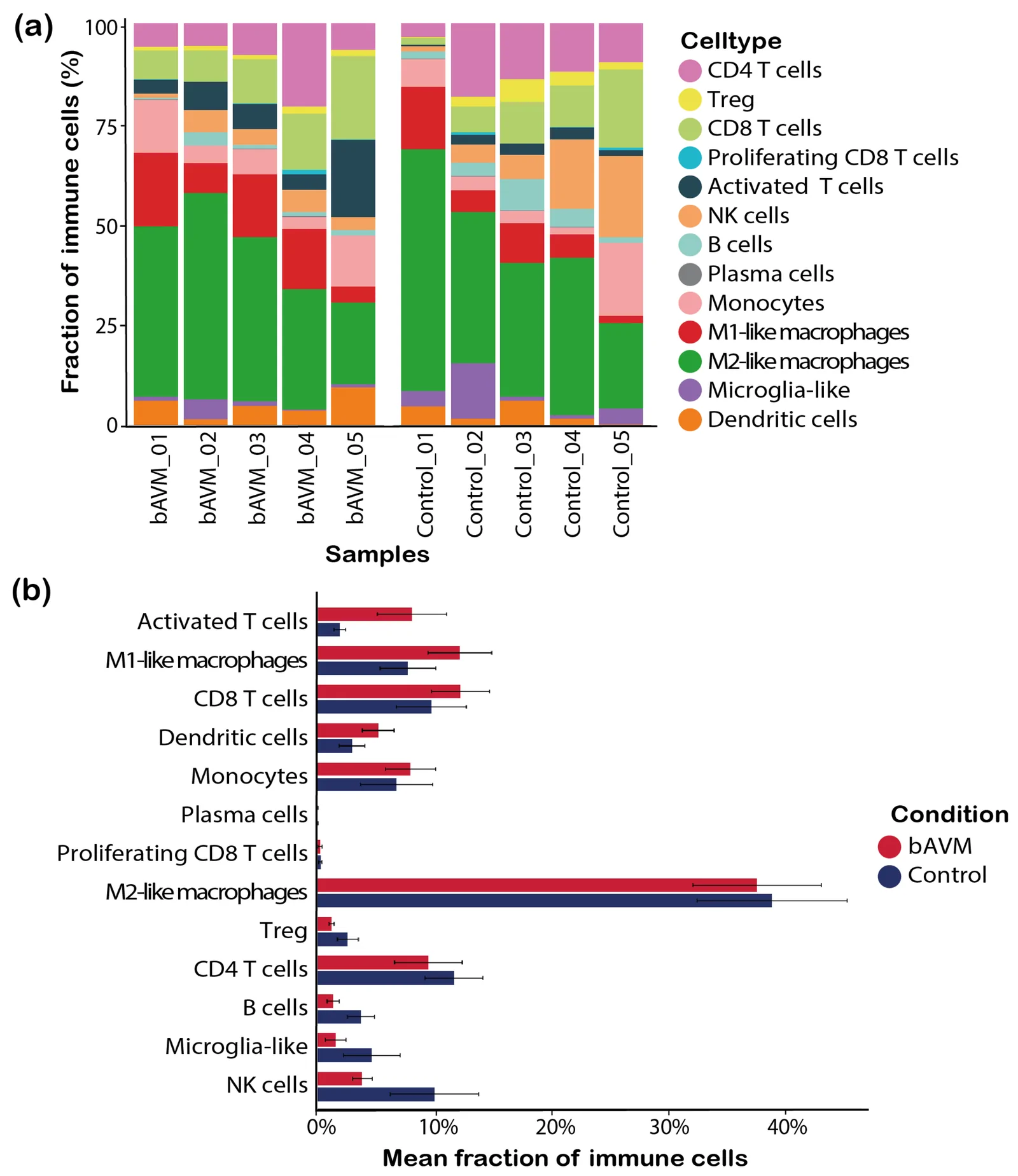
Immune-cell composition in bAVM and control brain vascular tissue. (**a**) Stacked bar plot showing the fraction of each immune-cell population within individual samples. Each bar represents one biological sample, and colors denote annotated immune-cell types. (**b**) Horizontal bar plot summarizing mean sample-level fractions of immune-cell populations in bAVM and control samples. Error bars represent the standard error of the mean. Abbreviations: Treg, regulatory T cells.

We next assessed immune cell composition in bAVMs and controls by calculating the fraction of each immune population per sample. Compared with controls, bAVM samples exhibited higher mean fractions of activated T cells (8.0% vs. 1.9%), M1-like macrophages (12.0% vs. 7.7%), CD8 T cells (12.1% vs. 9.6%), dendritic cells (5.2% vs. 3.0%), and monocytes (7.9% vs. 6.7%) (Figure 2). Among these, the increase in activated T cells showed the strongest signal (Δ = +6.1%, *p* = 0.012), although this did not remain significant after correction for multiple testing. Overall, these findings suggest a shift toward a myeloid and activated lymphoid immune landscape in bAVMs.

### 3.3. Angiogenesis- and Extracellular Matrix Remodeling-Related Transcriptional Programs Are Concentrated in Myeloid Cells and Increased in bAVMs

**Figure 3:**
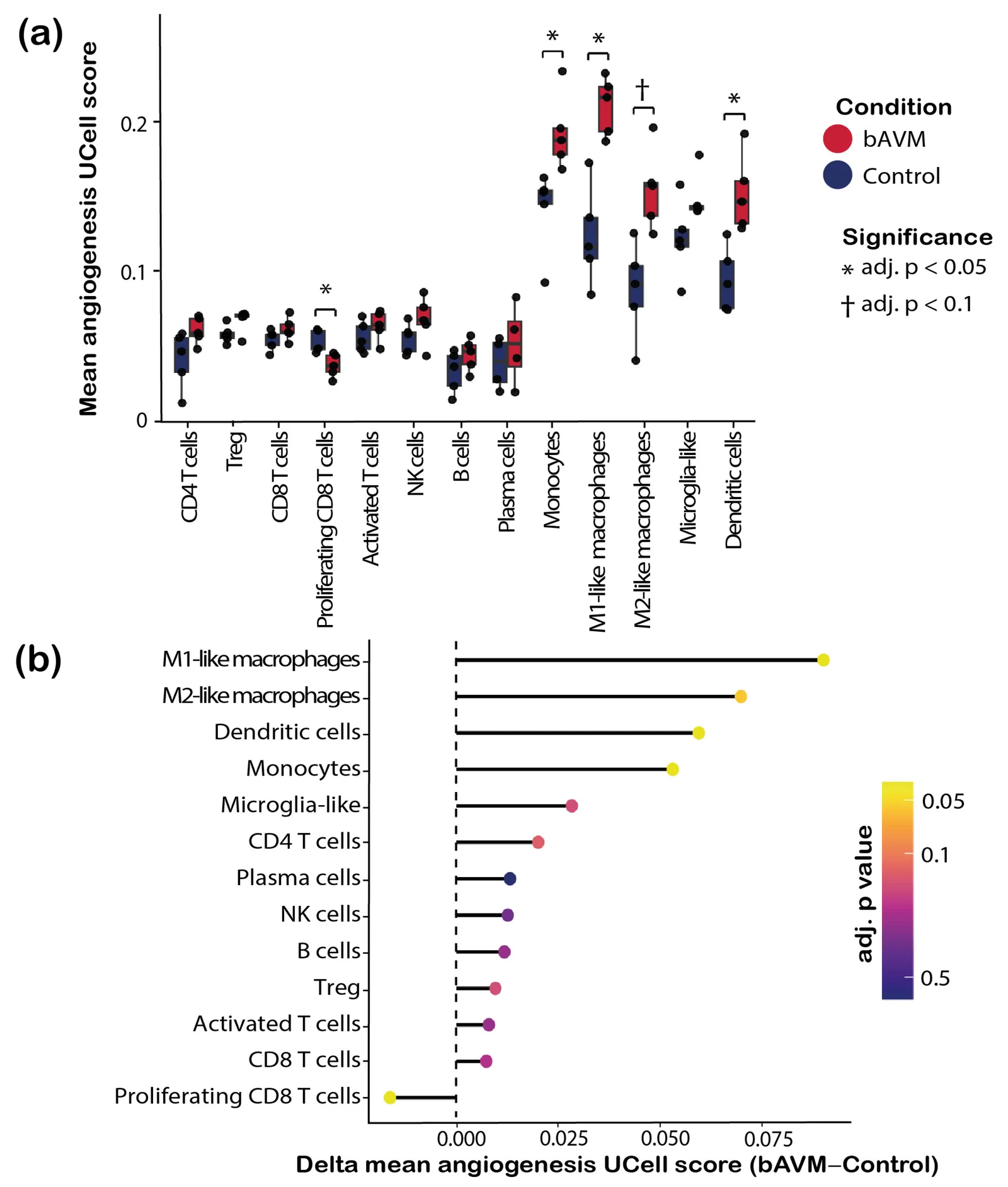
Angiogenesis-related transcriptional programs across immune cell populations in bAVMs. (**a**) Boxplots showing sample-level angiogenesis UCell scores across immune-cell populations in bAVM and control samples. Each point represents the mean program score for a given sample and cell type. (**b**) Plot summarizing the difference in mean angiogenesis score between bAVM and control samples for each immune-cell population.

To determine whether specific immune cell populations were associated with angiogenesis-related processes, we quantified angiogenesis program activity using UCell-based scoring [18]. Across the immune landscape, myeloid populations (i.e., monocytes, M1-like and M2-like macrophages, microglia-like cells and dendritic cells) exhibited consistently higher mean angiogenesis scores than lymphoid populations, indicating that angiogenesis-associated transcriptional activity is primarily concentrated within the myeloid compartment (Figure 3a).

When comparing bAVM and control samples within individual immune cell types, M1-like macrophages (0.22 vs. 0.13), monocytes (0.20 vs. 0.15), and dendritic cells (0.16 vs. 0.10) showed higher mean angiogenesis scores in bAVMs than in controls, whereas proliferating CD8 T cells showed a lower score (0.04 vs. 0.05). All four populations showed complete separation between bAVM and control samples and therefore they were tied at the four smallest ranks, resulting in the adjusted *p*-value 0.04. M2-like macrophages showed a comparable increase (0.16 vs. 0.09) that did not reach significance (adjusted *p* = 0.056; Figure 3b). The UCell angiogenesis score reflects the relative enrichment of predefined angiogenesis-associated genes, with higher values indicating stronger relative expression of angiogenic programs. Other myeloid and lymphoid populations displayed comparatively smaller differences. 

Next, we assessed ECM-associated transcriptional programs across immune cell populations. Similar to the angiogenesis analysis, ECM-related activity was predominantly observed in myeloid populations, whereas most lymphoid populations showed comparatively low scores (Figure S3a). Monocytes, microglia-like cells, and dendritic cells displayed higher mean ECM scores in bAVMs than in control samples, although these differences did not reach statistical significance after multiple testing correction (adjusted *p* = 0.09 for each). The largest positive shifts in ECM scores were observed in M2-like macrophages, followed by dendritic cells, M1-like macrophages, monocytes, and microglia-like cells (Figure S3b). In contrast, lymphoid populations showed comparatively modest differences, and proliferating CD8 T cells again exhibited a negative change in ECM score.

## 4. Discussion

In this study, we performed a single-cell transcriptomic analysis of immune cells derived from bAVMs and control brain vascular tissue to characterize the immune microenvironment of these lesions and to identify immune populations associated with vascular remodeling programs. Our findings demonstrate that the immune landscape of bAVMs shows a trend towards higher proportions of myeloid and activated lymphoid populations compared with control tissue. Further, transcriptional programs related to angiogenesis and, while not significant, extracellular matrix remodeling are predominantly concentrated in myeloid populations of bAVMs, particularly monocytes, macrophages, and dendritic cells.

### 4.1. Immune Composition in bAVMs Reflects Activation and Inflammatory Remodeling

We observed compositional shifts in immune cell populations between bAVM and control samples. bAVMs were enriched for activated T cells, CD8 T cells, M1-like macrophages, dendritic cells, and monocytes. While not significant after Benjamini–Hochberg correction, this shift may reflect disruption of normal immune homeostasis, whereas the expansion of activated immune cells may contribute to the dynamic remodeling of bAVMs.

### 4.2. Myeloid Cells Express Vascular Remodeling-Associated Transcriptional Programs in bAVMs

One of the central findings of this study is that the angiogenesis UCell score, which reflects angiogenesis-related transcriptional activity, is largely confined to the myeloid compartment of the immune landscape in bAVMs. Across immune populations, monocytes, M1-like macrophages, and dendritic cells displayed the highest angiogenesis program scores and showed the greatest differences between bAVM and control samples. This observation is consistent with prior histological and molecular studies reporting that macrophages regulate angiogenesis through the secretion of proangiogenic factors, matrix-degrading enzymes, and cytokines that influence endothelial cell behavior [9]. In the context of vascular malformations, such signals could contribute to abnormal vessel formation or maintenance. Our single-cell analysis extends these observations by demonstrating that angiogenesis-related transcriptional programs are not broadly distributed across immune cells but are concentrated in specific myeloid populations.

In addition to angiogenesis-related programs, we examined transcriptional signatures associated with ECM remodeling. Similar to the angiogenesis analysis, ECM-related activity was predominantly observed in myeloid populations. Although statistical significance was modest—likely reflecting the limited sample size, the large number of cell types tested, and correction for multiple comparisons—the directionality of ECM-related changes consistently pointed toward myeloid populations as the principal immunological contributors to ECM remodeling. Compared with ECM signatures, angiogenesis-related scores were more pronounced, which may reflect stronger or more consistently expressed transcriptional programs, whereas ECM-related processes are more heterogeneous, context-dependent, and potentially less robustly captured by the selected gene sets.

### 4.3. Implications for bAVM Pathophysiology

Together, these findings support a model in which the immune microenvironment of bAVMs may actively participate in the process of vascular remodeling. Myeloid cells appear to represent key cellular mediators linking inflammatory activity with angiogenic and potentially ECM-remodeling pathways. Such interactions may contribute to the persistence and structural instability of the bAVM nidus.

### 4.4. Limitations

Several limitations of this study should be acknowledged. First, the analysis was based on a publicly available dataset comprising a limited number of human samples. Although the single-cell resolution provides valuable insight into cellular heterogeneity, the relatively small number of biological replicates may limit the statistical power of some comparisons. Second, single-cell transcriptomic data capture transcriptional states but do not directly demonstrate functional activity of the identified pathways. Additional experimental validation will be required to confirm the biological roles of the identified immune populations. Third, after restriction to genes detectably expressed in immune cells, the scored gene sets were small (19 genes for angiogenesis and 8 for ECM remodeling). Scores that derived from few genes may show reduced robustness, particularly for the ECM remodeling module. Confirmation using larger or alternative gene sets, or targeted validation of individual mediators, would strengthen these findings. Further, the control tissue was obtained from patients undergoing surgery for drug-resistant epilepsy. Because epilepsy is itself associated with inflammation, blood–brain-barrier dysfunction, and microglial activation, this tissue may not represent a quiescent baseline. Such baseline activation would, however, be expected to reduce the apparent difference between bAVM and control tissue, suggesting that the immune activation reported here is likely conservative. Validation against non-inflamed control tissue will be an important next step. Additionally, the bAVM samples analyzed here were derived exclusively from unruptured, previously untreated lesions. While rupture status therefore does not confound the findings, it also means that they cannot be generalized to ruptured bAVMs. Beyond rupture status, the lack of angioarchitectural characteristics (lesion size, location, Spetzler–Martin grade, venous drainage pattern, and associated aneurysms) may influence the immune microenvironment. Therefore, confirmation in clinically and angioarchitecturally well-characterized cohorts will be an important next step. Future studies integrating spatial transcriptomics, proteomic analyses, and functional experimental models may help to further clarify the role of immune cells in bAVM biology. Finally, as with many single-cell datasets derived from dissociated tissue, certain immune populations such as neutrophils are underrepresented due to technical limitations of cell capture or low transcript counts.

## 5. Conclusions

This study provides a single-cell transcriptomic characterization of the immune microenvironment in human bAVMs. Angiogenesis- and ECM-related transcriptional activity was predominantly concentrated in myeloid populations, particularly monocytes, macrophages, and dendritic cells. These findings suggest that myeloid immune cells may play an important role in the vascular remodeling processes that characterize bAVMs.

## Data Availability

The original data analyzed in this study are publicly available at the UCSC Cell Browser repository: https://cells.ucsc.edu/?ds=adult-brain-vasc (accessed on 26 February 2026). All processed data objects and analysis code will be shared upon request.

## Supplementary Materials

The following supporting information can be downloaded at: https://www.mdpi.com/article/10.3390/brainsci16080811/s1, Figure S1: Initial clustering and lineage assignment of immune cells. (a) Uniform Manifold Approximation and Projection (UMAP) of all immune cells after quality control, doublet removal, and integration, colored by unsupervised clusters. (b) UMAP colored by major lineage annotation, distinguishing myeloid, lymphoid, and contaminant populations. (c) Heatmap showing average expression of selected marker genes across clusters, supporting lineage assignment. Figure S2: Lineage-specific reclustering and annotation of lymphoid and myeloid populations. (a) UMAP of lymphoid cells annotated into lymphoid cell types. (b) Heatmap showing average expression of selected marker genes across annotated lymphoid cell types. (c) UMAP of myeloid cells annotated into myeloid cell types. (d) Heatmap showing average expression of selected marker genes across annotated myeloid cell types. Abbreviations: Treg: regulatory T cells. Figure S3: ECM remodeling-related transcriptional programs across immune cell populations in bAVMs. (a) Boxplots showing sample-level ECM remodeling UCell scores across immune-cell populations in bAVM and control samples. Each point represents the mean program score for a given sample and cell type. (b) Plot summarizing the difference in mean ECM remodeling score between bAVM and control samples for each immune-cell population.
