# Supplementary material for: Single-Cell Analysis of Brain Arteriovenous Malformations Reveals Pro-Angiogenic Myeloid Programs
Source: Brain Sci. 2026 Jul 30;16(8):811. doi: 10.3390/brainsci16080811 (PMC13510454; doi:10.3390/brainsci16080811)
Supplement: Supplementary file 1 [file brainsci-16-00811-s001.zip › brainsci-4439141-supplementary.pdf]

Supplementary Materials

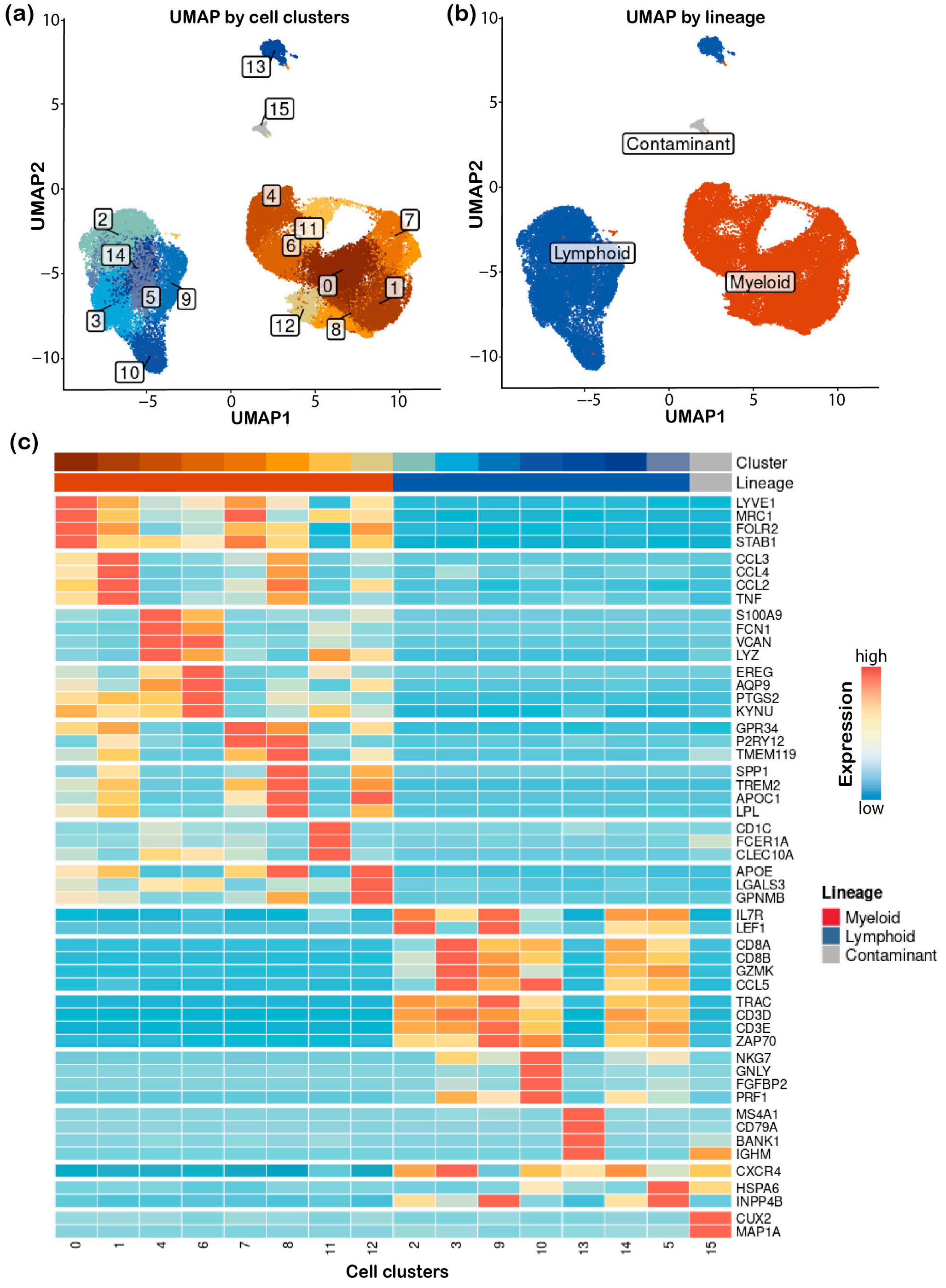

Figure S1: Initial clustering and lineage assignment of immune cells. (a) Uniform Manifold Approximation and Projection (UMAP) of all immune cells after quality control, doublet removal, and integration, colored by unsupervised clusters. (b) UMAP colored by major lineage annotation, distinguishing myeloid, lymphoid, and contaminant populations. (c) Heatmap showing average expression of selected marker genes across clusters, supporting lineage assignment.

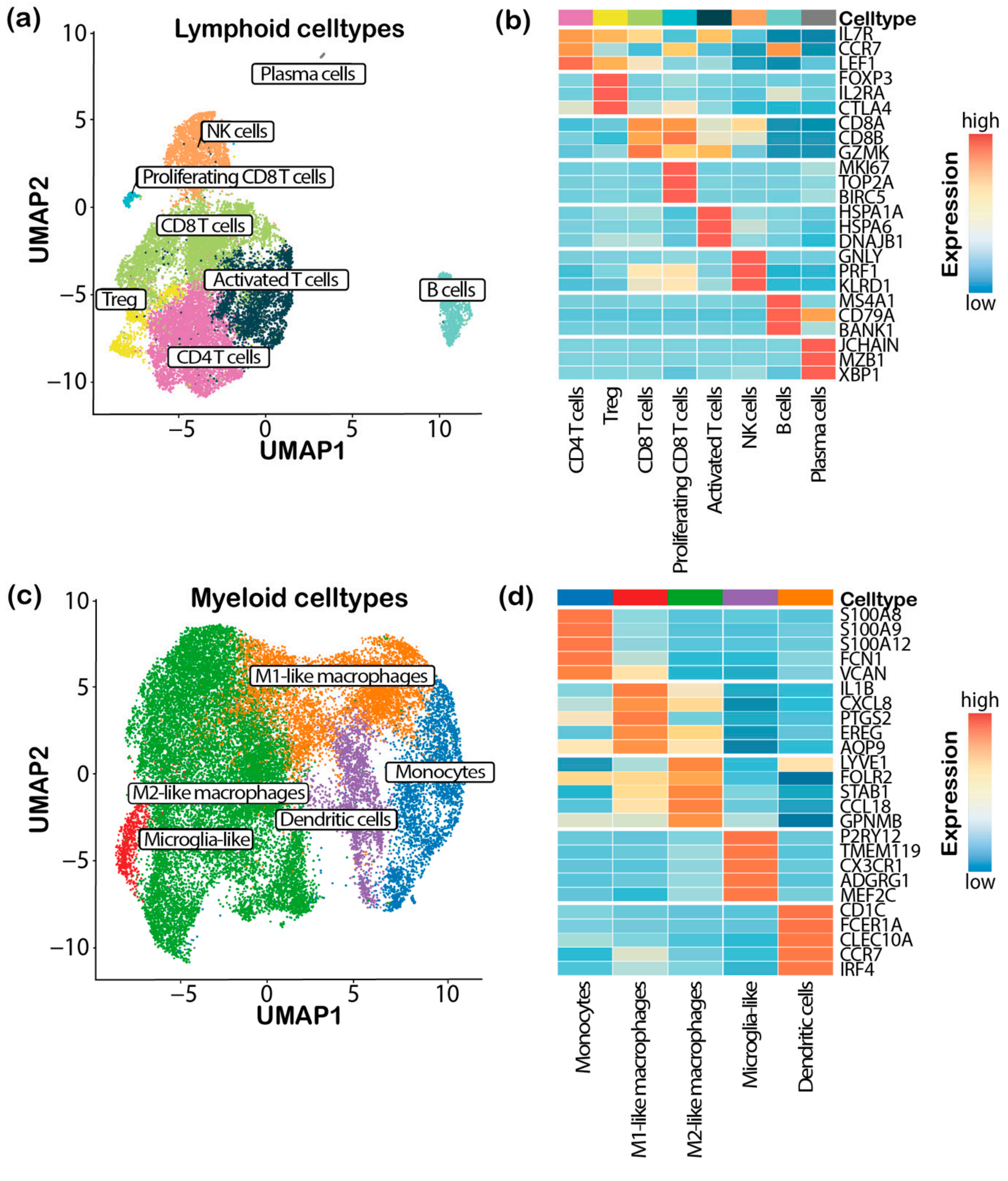

Figure S2: Lineage-specific reclustering and annotation of lymphoid and myeloid populations. (a) UMAP of lymphoid cells annotated into lymphoid cell types. (b) Heatmap showing average expression of selected marker genes across annotated lymphoid cell types. (c) UMAP of myeloid cells annotated into myeloid cell types. (d) Heatmap showing average expression of selected marker genes across annotated myeloid cell types. Abbreviations: Treg: regulatory T cells.

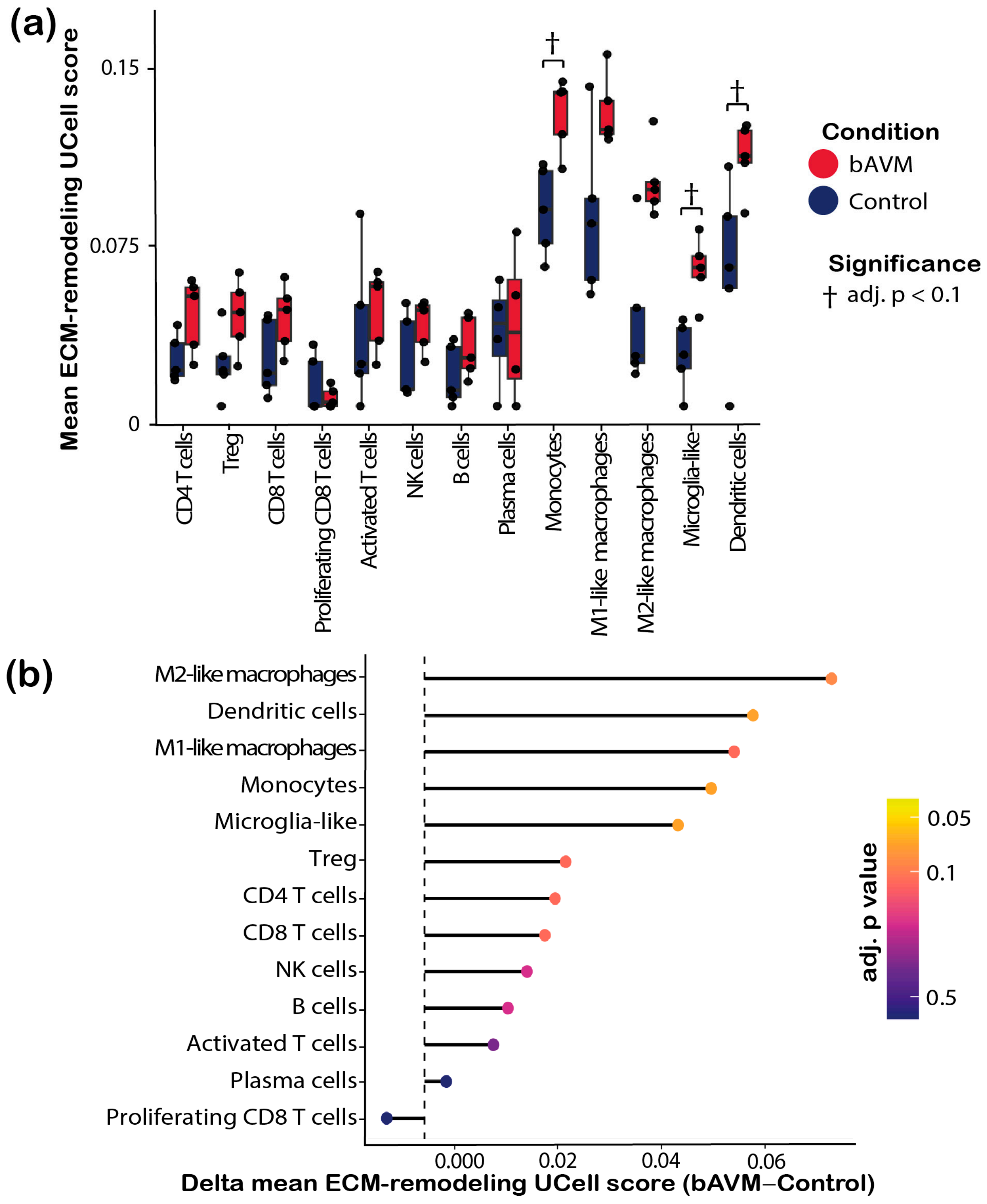

Figure S3: ECM remodeling-related transcriptional programs across immune cell populations in bAVMs. (**a**) Boxplots showing sample-level ECM remodeling UCell scores across immune-cell populations in bAVM and control samples. Each point represents the mean program score for a given sample and cell type. (**b**) Plot summarizing the difference in mean ECM remodeling score between bAVM and control samples for each immune-cell population.
